# Diagnostic Accuracy of the Biosynex CryptoPS Cryptococcal Antigen Semiquantitative Lateral Flow Assay in Patients with Advanced HIV Disease

**DOI:** 10.1128/JCM.02307-20

**Published:** 2020-12-17

**Authors:** Mark W. Tenforde, Timothée Boyer-Chammard, Charles Muthoga, Leabaneng Tawe, Thandi Milton, Ikanyeng Rulaganyang, Kwana Lechiile, Ivy Rukasha, Tshepo B. Leeme, Nelesh P. Govender, Julia Ngidi, Madisa Mine, Síle F. Molloy, Thomas S. Harrison, Olivier Lortholary, Joseph N. Jarvis

**Affiliations:** a Division of Allergy and Infectious Diseases, Department of Medicine, University of Washington School of Medicine, Seattle, Washington, USA; b Department of Epidemiology, University of Washington School of Public Health, Seattle, Washington, USA; c Institut Pasteur, CNRS, Molecular Mycology Unit, UMR2000, Paris, France; d Botswana-UPenn Partnership, Gaborone, Botswana; e Botswana Harvard AIDS Institute Partnership, Gaborone, Botswana; f National Institute for Communicable Diseases, a Division of the National Health Laboratory Service and School of Pathology, Faculty of Health Sciences, University of the Witwatersrand, Johannesburg, South Africa; g Botswana National Health Laboratory, Gaborone, Botswana; h Centre for Global Health, Institute for Infection and Immunity, St George's, University of London, London, United Kingdom; i Université de Paris, Centre d’Infectiologie Necker-Pasteur, APHP, IHU Imagine, Hôpital Necker Enfants Malades, Paris, France; j Department of Clinical Research, Faculty of Infectious and Tropical Diseases, London School of Hygiene and Tropical Medicine, London, United Kingdom; University of Iowa College of Medicine

**Keywords:** cryptococcal meningitis, CrAg, semiquantitative, HIV, lateral flow assay, Botswana, cryptococcal antigen, cryptococcosis, diagnostics

## Abstract

High cryptococcal antigen (CrAg) titers in blood are associated with subclinical meningitis and mortality in CrAg-positive individuals with advanced HIV disease (AHD). We evaluated a novel semiquantitative lateral flow assay (LFA), CryptoPS, that may be able to identify individuals with high CrAg titers in a cohort of AHD patients undergoing CrAg screening. In a prospective cohort of patients with AHD (CD4 cell count, ≤200/μl) receiving CD4 count testing, whole blood was tested for CrAg by CryptoPS and the IMMY LFA; the two assays were conducted by two different operators, each blind to the results of the other assay. The sensitivity, specificity, positive predictive value (PPV), and negative predictive value (NPV) of CryptoPS were assessed against the IMMY LFA as a reference. CryptoPS low-titer (T1 band) and high-titer (T2 band) results were compared with IMMY LFA titers obtained through serial dilution. A total of 916 specimens were tested. The sensitivity of the CryptoPS assay was 61.0% (25/41) (95% confidence interval [95% CI], 44.5 to 75.8%), its specificity was 96.6% (845/875) (95% CI, 95.1 to 97.7%), its PPV was 45.5% (95% CI, 32.0 to 59.4%), and its NPV was 98.1% (95% CI, 97.0 to 98.9%). All (16/16) CryptoPS false-negative results were obtained for samples with IMMY titers of ≤1:160. Of 29 patients (30 specimens) who tested positive by CryptoPS but negative by the IMMY LFA, none developed cryptococcal meningitis over 3 months of follow-up without fluconazole. Median CrAg titers were 1:20 (interquartile range [IQR], 0 to 1:160) in CryptoPS T1-positive samples and 1:2,560 (IQR, 1:1,280 to 1:10,240) in T2-positive samples. We conclude that the diagnostic accuracy of the CryptoPS assay was suboptimal in the context of CrAg screening, with poor sensitivity at low CrAg titers. However, the CryptoPS assay reliably detected individuals with high titers, which are associated with poor outcomes.

## Abstract (precis)

High cryptococcal antigen (CrAg) titers in blood are associated with subclinical meningitis and mortality in CrAg-positive individuals with advanced HIV disease (AHD). We evaluated a novel semiquantitative lateral flow assay (LFA), CryptoPS, that may be able to identify individuals with high CrAg titers in a cohort of AHD patients undergoing CrAg screening. In a prospective cohort of patients with AHD (CD4 cell count, ≤200/μl) receiving CD4 count testing, whole blood was tested for CrAg by CryptoPS and the IMMY LFA; the two assays were conducted by two different operators, each blind to the results of the other assay.

## INTRODUCTION

Cryptococcal meningitis (CM) remains a major cause of mortality in persons living with HIV (PLWH), causing an estimated 15% of HIV-related deaths worldwide (1). In individuals with advanced HIV disease (AHD), detection of cryptococcal antigen (CrAg) in the blood has been shown to precede the development of CM (2, 3). CrAg screening of patients with AHD starting antiretroviral therapy (ART), with targeted preemptive fluconazole for CrAg-positive patients, has been shown to reduce incident CM and all-cause mortality (4) and is recommended by the World Health Organization (WHO) for those starting or reinitiating ART with a CD4 T-cell count of <200/μl (5).

Preemptive fluconazole therapy for CrAg-positive individuals reduces, but does not completely eliminate, excess mortality (6); as many as one-third of asymptomatic CrAg-positive patients with AHD have disseminated central nervous system (CNS) disease and may require more-potent antifungal regimens for effective treatment (5–7). Higher CrAg titers (>1:160) are associated with an increased risk of CNS disease and death in CrAg-positive individuals (7–9). Titers can be assessed through serial dilution using commercial assays, but this requires extended labor and additional costs, making it impractical in resource-limited settings. Semiquantitative CrAg assays may provide a simpler means of stratifying risk for CrAg-positive patients with AHD and guiding differentiated treatment strategies.

**FIG 1:**
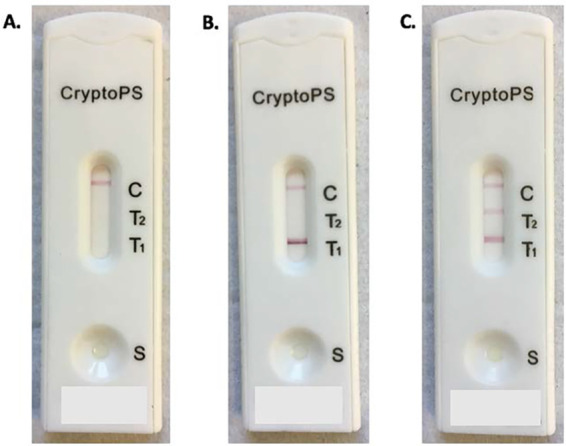
CryptoPS semiquantitative assay. Twenty microliters of a blood specimen is added to the collection well of the cassette, followed by 3 drops of sample diluent, with the result read at 10 min. (A) Negative result (control band positive); (B) :Low-titer positive result (T1 and control bands positive); (C) High-titer positive result (T1, T2, and control bands positive). Patient initials and study identification codes written on the bottoms of the test strips have been concealed to ensure the anonymity of the research participants.

CryptoPS (Biosynex, Strasbourg, France) is a semiquantitative immunochromatographic assay that detects all Cryptococcus neoformans*/*Cryptococcus gattii serotypes and provides results within 10 min. The assay has a control band, a qualitative T1 band for CrAg positivity, and a semiquantitative T2 band indicating a “high” CrAg titer (Fig. 1). Just two limited diagnostic evaluations have been reported to date (10, 11). In a small study of patients with CD4 cell counts of <100/μl in Cameroon, CryptoPS had good agreement with a commercial lateral flow assay (LFA) (IMMY Diagnostics, Norman, OK, USA) and an enzyme immunoassay (EIA) (Premier CrAg; Meridian Bioscience, Inc., Newtown, OH, USA), with a median reciprocal EIA titer of >1:160 correlating with T2 band positivity, although the sample size, which included just 14 serum CrAg-positive patients, limited estimates of precision (10). A second study testing 99 archived serum samples selected from a cohort of HIV-positive Ugandans undergoing CrAg screening reported similar findings, with reasonable agreement between the CryptoPS and IMMY LFA and median CrAg titers of 1:320 for those with T2 band positivity (11); however, this study was also limited by a small sample size. CryptoPS was not tested on whole-blood samples, likely to be beneficial for point-of-care testing, in either study, and the assay has not been evaluated prospectively and under routine care conditions.

We investigated the diagnostic performance of the CryptoPS assay in a prospective CrAg screening cohort in Botswana, evaluating test accuracy (sensitivity, specificity, positive predictive value [PPV], and negative predictive value [NPV]) against a validated reference standard. To determine titer cutoffs for the CryptoPS T2 band (high titers), we compared CryptoPS readings with IMMY CrAg titer results by serial dilution.

## MATERIALS AND METHODS

From January 2018 to January 2019, we performed real-time CrAg testing on EDTA whole-blood samples from a sequential cohort of individuals with CD4 cell counts of ≤200/μl undergoing routine reflex CrAg screening at the Botswana-Harvard HIV Reference Laboratory (BHHRL). The BHHRL performs all CD4 testing for 27 ART clinics and a central referral hospital located around Gaborone. The full cohort has been described in detail elsewhere (12); briefly, CD4 T-cell count testing was performed using flow cytometry (FACSCalibur; Becton, Dickinson and Company, Franklin Lakes, NJ, USA); residual EDTA blood from sequential samples with CD4 cell counts of ≤200/μl was screened for CrAg in real time using the IMMY LFA (IMMY, Norman, OK, USA) as a validated reference standard (13, 14). CryptoPS testing was performed by a second trained operator blind to the IMMY LFA results. Results were interpreted by visual reading as negative (only the control band was visualized), T1 band positive (with the control and T1 bands visualized by the operator), or T2 band positive (control, T1, and T2 bands visualized), as per manufacturer’s instructions (Fig. 1).

Since the CryptoPS is not registered for diagnostic use in Botswana, all clinical management decisions were based on IMMY LFA results. IMMY LFA-positive patients were managed according to the Botswana National HIV Management Guidelines (15). Individuals who tested positive by CryptoPS but negative by the IMMY LFA were contacted by the study team for retesting with the IMMY LFA and were monitored for 3 months in order to be evaluated for the development of incident CM, but they did not receive antifungal therapy unless the repeat IMMY LFA results were positive. Three-month outcomes in these individuals with positive CryptoPS results but negative IMMY LFA results were assessed through telephone contact, review of a national electronic medical records system, review of clinical notes, or further contact with clinical providers as needed.

At the end of the study period, IMMY LFA titers were determined by serial dilution of plasma samples stored at −80°C. To enable further validation of the CryptoPS semiquantitative results, we used both CryptoPS and the IMMY LFA to test an additional collection of 141 archived known CrAg-positive plasma samples from (i) patients in a CrAg screening cohort from 2015 to 2016 (8) and (ii) patients with CM enrolled in a completed phase II clinical trial evaluating induction therapies (16). All CrAg testing was performed by laboratory scientists blind to previous testing results.

Finally, to better characterize samples with discordant CryptoPS and IMMY LFA test results, we retested all discordant samples with Meridian’s CrAg EIA (Meridian Bioscience, Cincinnati, OH) and IMMY’s EIA (IMMY, Norman, OK) at the National Institute for Communicable Diseases (NICD) in Johannesburg, South Africa. This study was approved by institutional review boards at the Botswana Ministry of Health and Wellness, the University of Botswana, Princess Marina Hospital, and the University of Pennsylvania.

### Statistical analysis.

In the prospective CrAg screening cohort, we determined the sensitivity, specificity, PPV, and NPV of the CryptoPS assay with 95% confidence intervals (95% CI) using the IMMY LFA assay as a reference standard. Using all CrAg-positive samples from the prospective screening cohort, plus the additional archived CrAg-positive samples, we evaluated the median and interquartile range (IQR) of dilutional IMMY LFA CrAg titer values in samples with CryptoPS that were T1 band and T2 band positive, and we displayed the results graphically.

Laboratory findings, including the results of cryptococcal EIA testing, were described for samples with discordant CryptoPS-LFA results, and clinical outcomes were reported for individuals with negative IMMY LFA but positive CryptoPS results. Analyses were performed using Stata, version 16 (College Station, TX, USA).

## RESULTS

### Sensitivity and specificity of CryptoPS in a sequential cohort of individuals with CD4 cell counts of ≤200/μl.

A total of 916 sequential blood samples from 870 unique patients (of whom 46 had repeat CD4 testing and reflex CrAg screening [Table 1]) were tested with the IMMY LFA and the CryptoPS CrAg assay; the median age was 39 years (interquartile range [IQR], 33 to 46 years), 58.4% (507/868; 2 missing data) were male, and the median CD4 cell count was 142/μl (IQR, 85 to 174 cells/μl). By use of IMMY LFA testing, 41 of the 916 samples were CrAg positive (4.5% [95% confidence interval {CI}, 3.2 to 6.0%]) with a median CrAg titer of 1:80 (IQR, 1:20 to 1:960). Using the IMMY LFA as the reference standard, the sensitivity of CryptoPS was 61.0% (95% CI, 44.5 to 75.8%), and its specificity was 96.6% (95% CI, 95.1 to 97.7%), yielding a PPV of 45.5% (95% CI, 32.0 to 59.4%) and an NPV of 98.1% (95% CI, 97.0 to 98.9%) in our cohort (Table 2).

**TABLE 1:** Baseline characteristics of study participants^*a*^

Group and variable	Value^*b*^
Sequential cohort of individuals with CD4 cell counts of ≤200/μl screened for CrAg (916 samples from 870 individuals)^*c*^	
Age (yr) (IQR)^*d*^	39 (33–46)
% male^*d*^	58 (507/868^*e*^)
CD4 cell count/μl (IQR)^*f*^	142 (86–174)
% (no.) positive for cryptococcal antigenemia^*f*^^,^^*g*^	4.5 (41)
% on ART^*f*^	75 (691/916)
% with prior cryptococcal meningitis^*d*^	1 (13/870)
Additional individuals included in titer validation cohort (all CrAg positive [*n* = 141])^*g*^	
Age (yr) (IQR)	38 (32–44)
% male	58 (81/139^*e*^)
CD4 cell count/μl (IQR)	31 (12–63)
% (no.) positive for cryptococcal antigenemia^*g*^	100 (141)

a ART, antiretroviral therapy; CrAg, cryptococcal antigen; IQR, interquartile range; LFA, lateral flow assay.

b Except where otherwise indicated, values are given as the median (IQR) or as a percentage (number of individuals with the characteristic/total number).

c A total of 916 samples from 870 unique patients were tested; 46 individuals had repeat samples.

d Age, sex, and prior cryptococcal meningitis data are reported for the 870 individuals in the CrAg screening cohort at the time of their first CrAg test.

e Data were missing for two individuals.

f CD4 cell counts, IMMY CrAg LFA results, and ART status at the time of testing (some patients were tested twice during screening period) are shown; of 226 patients not on ART at date of CD4 testing, 17% (39) had defaulted from ART and 83% (187) had no history of ART use.

g CrAg results obtained using the IMMY CrAg lateral flow assay.

**TABLE 2:** Sensitivity, specificity, and positive, and negative predictive values of the CryptoPS semiquantitative assay versus the conventional IMMY LFA^*a*^

Result by CryptoPS	No. of samples with the following result by the IMMY LFA:		Total no. of samples with the indicated result by CryptoPS	CryptoPS accuracy (% [95% CI])			
	Positive	Negative		Sensitivity	Specificity	PPV	NPV
Positive	25	30	55				
Negative	16	845	861				
Total	41	875	916	61.0 (44.5–75.8)	96.6 (95.1–97.7)	45.5 (32.0–59.4)	98.1 (97.0–98.9)

a Tests were performed on whole-blood samples. PPV, positive predictive value; NPV, negative predictive value; CI, confidence interval; LFA, lateral flow assay.

**FIG 2:**
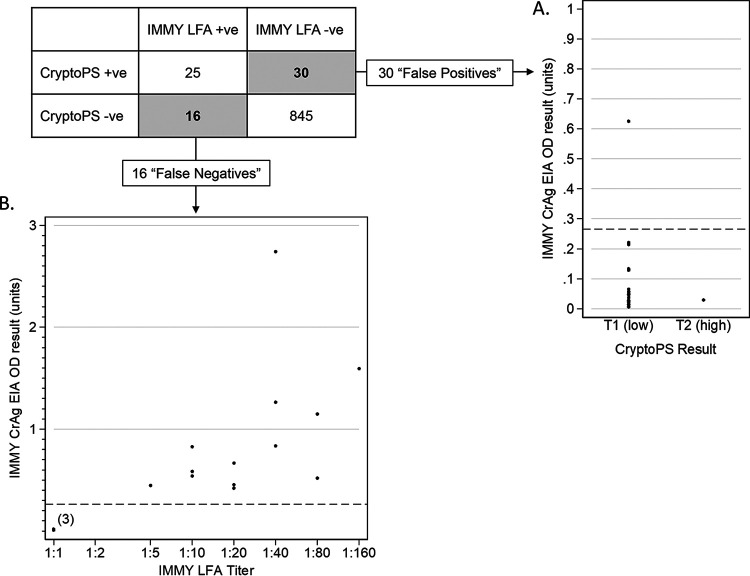
Discordant CryptoPS and IMMY LFA results. (A) CryptoPS “false-positive” results with secondary testing using the IMMY EIA; 29/30 samples tested negative on the IMMY EIA at an optical density below the positive cutoff, and 1 sample tested positive at an optical density of 0.625. All samples tested negative using the Meridian EIA. (B) CryptoPS “false-negative” results with IMMY LFA dilutional titer and IMMY EIA optical density; 16/16 samples had an IMMY LFA titer of ≤1:160, and 3 tested negative on the IMMY EIA at an optical density below the positive cutoff, all 3 were IMMY LFA positive only in undiluted samples, indicating very low titers.

The CryptoPS assay was positive in 55/916 (6.0% [95% CI, 4.6 to 7.7%]) of samples; 25/55 (45.5%) were IMMY LFA positive and were classified as “true positives” (18 T1 band and 7 T2 band). The remaining 30/55 (54.5%) CryptoPS-positive samples were IMMY LFA negative and were classified as “false positives” (29 T1 band and 1 T2 band). On confirmatory testing, all 30 of the CryptoPS false-positive results were Meridian EIA negative, and 29/30 were IMMY EIA negative, with one low-level positive (Fig. 2a). Sixteen samples were IMMY LFA positive but CryptoPS negative and were classified as “false negatives”; all had IMMY LFA CrAg titers of ≤1:160, and 13 of the 16 had titers of ≤1:40. Three of the 16 were LFA positive only at the lowest dilution tested (1:2) and negative on the IMMY EIA (Fig. 2b).

Detailed characteristics of the patients with the 30 false-positive CryptoPS results are shown in Table 3. The 30 “false-positive” CryptoPS samples were from 29 patients (one had two tests); 79% (23/29) received repeat IMMY LFA testing, of whom 100% (23/23) were CrAg negative. Most (97% [28/29]) were on ART at the time of CrAg screening, and none received preemptive fluconazole. None (0/29) were diagnosed with cryptococcal meningitis in the 3 months following CD4 testing and reflex CrAg screening, and 28/29 were alive at the completion of follow-up. One patient died 5 days after the date of CD4 testing after presenting to hospital with a clinical diagnosis of a lower respiratory tract infection; no central nervous system infection was suspected, and the patient did not receive a lumbar puncture as part of his diagnostic evaluation. The patient’s CryptoPS result was T1 band positive, with negative IMMY LFA, IMMY EIA, and Meridian EIA results.

**TABLE 3:** Clinical characteristics and outcomes of patients with false-positive CryptoPS test results on whole-blood specimens^*a*^

Case	Age (yr)	Sex	CD4 count, cells/μl	On ART	Prior CM	Repeat IMMY LFA	Received antifungal therapy	Incident CM within 3 mo	Death within 3 mo	Comments
1	53	Male	169	Yes	No	Negative	No	No	No	
2	65	Male	106	Yes	No	Negative	No	No	No	
3	40	Female	25	Yes	No	Negative	No	No	No	
4	48	Female	145	Yes	No	Negative	No	No	No	
5	50	Female	106	Yes	No	Negative	No	No	No	
6	24	Female	44	Yes	No	Negative	No	No	No	
7	31	Female	83	Yes	No	Negative	No	No	No	
8	37	Female	161	Yes	No	Negative	No	No	No	
9	41	Male	115	Yes	No	Negative	No	No	No	
10	7	Male	197	Yes	No	Negative	No	No	No	
11	19	Male	143	Yes	No	Negative	No	No	No	
12	43	Female	158	Yes	No	Negative	No	No	No	
13	41	Male	32	Yes	No	Negative	No	No	No	
14	19	Female	147	Yes	No	Negative	No	No	No	
15	24	Male	134	Yes	No	Negative	No	No	No	
16	32	Male	149	Yes	No	Negative	No	No	No	
17	36	Female	103	Yes	No	Negative	No	No	No	
18	48	Male	194	Yes	No	Negative	No	No	No	
19	42	Male	106	Yes	No	Negative	No	No	No	
20	32	Female	68	Yes	No	Negative	No	No	No	
21	52	Female	193	Yes	No	Negative	No	No	No	
22^*b*^	45	Male	98	Yes	No	Negative	No	No	No	Lumbar puncture and CSF testing for cryptococcus (by CSF India ink and culture) 31 days after HIV test date; all CSF tests negative
23	33	Male	86	Defaulted	No	Negative	No	No	No	Restarted ART; CD4 increased to 156 cells/μl, and HIV viral load was 652 copies/μl 133 days after first CD4 test
24	39	Female	83	Yes	No	Not performed	No	No	No	Moved from Gaborone and unable to repeat IMMY LFA; reestablished care in Gaborone with HIV clinic visit 308 days after CrAg test; no cryptococcal meningitis diagnosed during follow-up.
25	39	Male	125	Yes	No	Not performed	No	No	No	Unable to contact for repeat CrAg testing; attended HIV clinic visit 287 days after CrAg test; no cryptococcal meningitis diagnosed during follow-up
26	20	Female	190	Yes	No	Not performed	No	No	No	Unable to contact for repeat CrAg testing; HIV clinic visit 363 days after CrAg test; no cryptococcal meningitis diagnosed during follow-up
27	Unknown	Male	171	Yes	No	Not performed	No	No	No	Participant unable to meet with study team for retesting but asymptomatic; repeat CD4 testing at 61 days from first test showed CD4 of 271 cells/μl and HIV viral load of <400 copies/ml
28	29	Female	125	Yes	No	Not performed	No	No	No	Unable to contact patient; was seen in clinic 37 days after CD4 test date and was stable, without reported symptoms and with an HIV viral load of <400 copies/ml; no subsequent laboratory evaluation for CM or documentation of death from national death registry
29	51	Male	32	Yes	No	Not performed	No	No	Yes	Presented to referral hospital Emergency Department 5 days after CD4 and CrAg test date; vomiting, anorexia, wasting with dehydration. Admission diagnosis of pneumonia and/or pulmonary tuberculosis, anemia, and renal failure. Died the following day; no clinical evidence of meningitis and no lumbar puncture and CSF evaluation performed; unlikely to have died from CM.

a ART, antiretroviral therapy; CrAg, cryptococcal antigen; CM, cryptococcal meningitis; CSF, cerebrospinal fluid; LFA, lateral flow assay.

b Patient had two positive CryptoPS test results.

Given the relatively low sensitivity and specificity of the CryptoPS assay on EDTA whole blood, we repeated CryptoPS testing on frozen plasma samples after study completion (with operators blind to the previous results), which yielded results very similar to those of real-time whole blood testing for these specimens (see Table S1 in the supplemental material). We also repeated the analysis restricted to individuals with CD4 cell counts of ≤100/μl; in this subgroup, the sensitivity of CryptoPS was 83.3% (95% CI, 58.6 to 96.4%), and its specificity was 96.5% (95% CI, 93.5 to 98.4%) (Table S2).

### Relationship between CryptoPS bands and dilutional CrAg titers.

**FIG 3:**
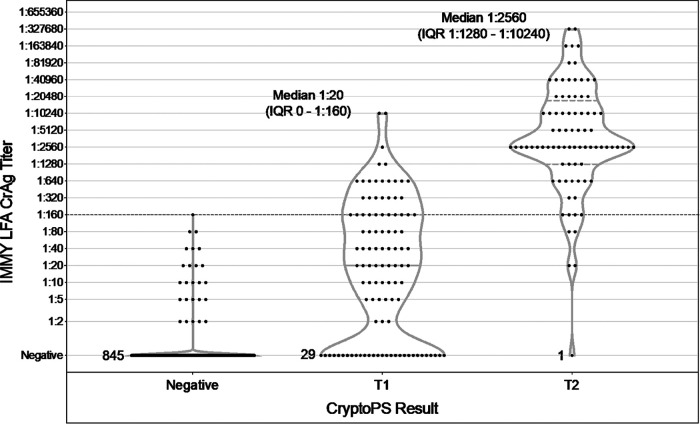
Comparison of CryptoPS titer results with dilutional titers obtained using the IMMY LFA. The violin plots show the distribution of dilutional CrAg titers in CryptoPS-negative, T1 (low-titer), and T2 (high-titer) samples, comprising 916 prospectively screened samples and 141 additional archived CrAg-positive plasma samples. Twenty-five CryptoPS-negative samples (16 from the prospective cohort and 9 archived samples) were positive on IMMY testing, all at titers of ≤1:160. Twenty-nine CryptoPS T1-positive samples and 1 CryptoPS T2-positive sample were IMMY LFA negative. Median titers in the T1 and T2 groups with interquartile ranges are given. Note that in the treatment trial from which some of the archived samples were taken, the maximum dilutional titer measured was 1:2,560; hence, some titers at this level may have been higher if further dilutions had been performed.

When the samples from the sequential CrAg screening cohort were combined with 141 archived IMMY LFA CrAg-positive blood samples, 870 samples were CryptoPS negative, 102 were T1 band positive, and 84 were T2 band positive (one CryptoPS T2-positive individual in the sequential cohort did not have sufficient residual plasma for titration). The median IMMY LFA titer of the T1 band-positive samples was 1:20 (IQR, LFA negative to 1:160). The median titer of the CryptoPS T2 band-positive samples was 1:2,560 (IQR, 1:1,280 to 1:10,240); 79/84 (94%) T2-positive samples had titers of ≥1:160 (Fig. 3).

## DISCUSSION

In this prospective evaluation of the novel semiquantitative CryptoPS CrAg LFA for HIV-seropositive individuals with CD4 cell counts of ≤200/μl who were undergoing CrAg screening, we found suboptimal assay sensitivity of CryptoPS compared to an established CrAg LFA (IMMY). The lower sensitivity of CryptoPS was exclusively due to false-negative test results on blood samples with low CrAg titers; all samples with titers of >1:160 were detected by the assay.

Our study adds to two small prior diagnostic validation studies of the CryptoPS assay. A study of stored serum samples from Cameroon found a test sensitivity of 78% (11/14) against the IMMY LFA (10). The median CD4 cell count of samples in this study was lower than that in our cohort (44 cells/μl versus 142 cells/μl), likely resulting in a higher median CrAg titer (1:160 versus 1:80) and contributing to slightly higher observed sensitivity (12). A second study of 99 cryopreserved serum samples from a Ugandan CrAg screening cohort found a CryptoPS assay sensitivity of 88% (50/57 [95% CI, 76% to 95%]) and a specificity of 95% (40/42) (95% CI, 84% to 99%) against the IMMY LFA; however, only 65% of samples with an IMMY LFA dilutional titer of ≤1:20 were positive on the CryptoPS assay (11). Similarly, in our prospective evaluation, all (16/16) false-negative whole-blood CryptoPS results were on samples with CrAg titers of ≤1:160, 13 of which (81%) had titers of ≤1:40. These patients may be at relatively low risk for progression to meningitis in the absence of preemptive fluconazole, assuming prompt initiation or continuation of effective ART (17).

While the CryptoPS assay had suboptimal overall sensitivity, the T2 band performed well in discriminating samples that had high CrAg titers with greater risk of meningitis and/or failure of preemptive fluconazole therapy (6), with 79/84 (94%) of T2 band-positive samples from stored blood found to have titers of ≥1:160 by serial dilution. The assay may have clinical utility in recognizing CrAg-positive patients who require further diagnostic evaluation for meningitis and enhanced antifungal treatment, albeit at the expense of missing a proportion of patients with lower CrAg titers who are still likely to benefit from standard preemptive fluconazole treatment (18). To avoid missing these individuals with lower titers, CryptoPS could be used as a second test, performed after an initial IMMY LFA screen, to identify the highest-risk individuals with T2 bands for enhanced care. The ease of use and interpretation of the CryptoPS tests may make this an attractive strategy in some settings.

We found moderate specificity of the CryptoPS assay, in keeping with the previous study from Uganda, but with greater estimated precision given our larger sample size (11). We carefully followed the 29 patients with presumed false-positive CryptoPS results (a positive CryptoPS result and a negative IMMY LFA result) for at least 3 months. All patients (23/29) with repeat IMMY LFA testing again tested negative; all were negative with secondary testing using the Meridian EIA, and all but one with the IMMY EIA. None developed meningitis despite receiving no antifungal treatment, supporting the classification of these results as false positive. Further, nearly all these patients were confirmed to be alive over follow-up; one patient died shortly after screening, but for causes likely unrelated to cryptococcal disease. Given a relatively low prevalence of cryptococcal antigenemia in patients with AHD (19), the positive predictive value of the CryptoPS assay in the prospective cohort was below 50%, with more false-positive than true-positive results.

Our study is subject to several limitations. First, *Cryptococcus* sp. isolates have significant serotype diversity within Southern Africa, which may impact test sensitivity (20, 21). Although the CryptoPS assay can detect all Cryptococcus neoformans/Cryptococcus gattii serotypes, different genotypic and phenotypic characteristics of local fungi could potentially have impacted test performance. Second, although the IMMY LFA and the CryptoPS test were read by trained laboratory operators blind to the results of the other assay, we did not have two independent reviewers read the CryptoPS assay to evaluate inter-rater reliability. However, we repeated CryptoPS testing on the same stored plasma specimens with similar test results, and all IMMY LFA-positive samples underwent further testing through serial dilution, confirming positive results, so the findings are highly likely to be valid.

In summary, the CryptoPS assay provides rapid semiquantitative CrAg test results on whole-blood specimens and good discrimination for high-titer results. Limitations in sensitivity and specificity may limit the clinical use of this assay in cryptococcal antigen-screening programs, which typically aim to detect individuals with relatively low cryptococcal antigen burdens. These limitations may be less marked when one is testing individuals with suspected cryptococcal meningitis, who have a higher pretest probability of infection and higher blood CrAg titers.

## Supplementary Material

Supplemental file 1
