# Supplementary material for: Diagnostic Accuracy of the Biosynex CryptoPS Cryptococcal Antigen Semiquantitative Lateral Flow Assay in Patients with Advanced HIV Disease
Source: J Clin Microbiol. 2020 Dec 17;59(1):e02307-20. doi: 10.1128/JCM.02307-20 (PMC7771453; doi:10.1128/JCM.02307-20)
Supplement: Supplemental file 1 [file JCM.02307-20-s0001.pdf]

Supplementary Materials

Diagnostic accuracy of the Biosynex CryptoPS cryptococcal antigen semi-quantitative lateral flow assay in patients with advanced HIV disease

Table S1. Page 2

Table S2. Page 3

Table S1. Sensitivity, specificity, positive, and negative predictive value of the CryptoPS semi-quantitative (SQ) LFA versus the conventional IMMY LFA test, frozen plasma samples.

		**IMMY LFA +ve**	**IMMY LFA -ve**	Total
	**CryptoPS +ve**	26	47	73
	**CryptoPS -ve**	14	823	837
	Total	40	870	910
Sensitivity		65.0%	95% CI 48.3% - 79.4%	
Specificity		94.6%	95% CI 92.9% - 96.0%	
Positive predictive value		35.6%	95% CI 24.7% - 47.7%	
Negative predictive value		98.3%	95% CI 97.2% - 99.1%	

CI = confidence interval; LFA = lateral flow assay; SQ = semi-quantitative, +ve = positive; -ve = negative

Table S2. Sensitivity, specificity, positive, and negative predictive value of the CryptoPS semi-quantitative (SQ) LFA versus the conventional IMMY LFA test, restricted to individuals with CD4 cell counts ≤100 cells/µL.

		**IMMY LFA +ve**	**IMMY LFA -ve**	Total
	**CryptoPS +ve**	15	9	24
	**CryptoPS -ve**	3	251	254
	Total	18	260	278
Sensitivity		83.3%	95% CI 58.6% - 96.4%	
Specificity		96.5%	95% CI 93.5% - 98.4%	
Positive predictive value		62.5%	95% CI 40.6% - 81.2%	
Negative predictive value		98.8%	95% CI 96.6% - 99.8%	

CI = confidence interval; LFA = lateral flow assay; SQ = semi-quantitative, +ve = positive; -ve = negative
